# Proteomics analysis of serum from thymoma patients

**DOI:** 10.1038/s41598-023-32339-4

**Published:** 2023-03-29

**Authors:** Jiaduo Li, Guoyan Qi, Yaling Liu

**Affiliations:** People’s Hospital of Shijiazhuang Affiliated to Hebei Medical University, Shijiazhuang, Hebei China

**Keywords:** Tumour biomarkers, Cancer, Oncology

## Abstract

Thymoma is the most common malignant tumor in thymic epithelial tumors (TETS). This study aimed to identify the changes in serum proteomics in patients with thymoma. Proteins were extracted from twenty patients with thymoma serum and nine healthy controls and prepared for mass spectrometry (MS) analysis. Data independent acquisition (DIA) quantitative proteomics technique was used to examine the serum proteome. Differential proteins of abundance changes in the serum were identified. Bioinformatics was used to examine the differential proteins. Functional tagging and enrichment analysis were conducted using the Gene Ontology (GO) and Kyoto Encyclopedia of Genes and Genomes (KEGG) databases. The string database was used to assess the interaction of different proteins. In all, 486 proteins were found in all samples. There were differences in 58 serum proteins between patients and healthy blood donors, 35 up-regulated and 23 down-regulated. These proteins are primarily exocrine and serum membrane proteins involved in controlling immunological responses and antigen binding, according to GO functional annotation. KEGG functional annotation showed that these proteins play a significant role in the complement and coagulation cascade and the phosphoinositide 3-kinase (PI3K)/protein kinase B (AKT) signal pathway. Notably, the KEGG pathway (complement and coagulation cascade) is enriched, and three key activators were up-regulated: von willebrand factor (VWF), coagulation factor v (F5) and vitamin k-dependent protein c (PC). Protein–protein interaction (PPI) analysis showed that six proteins ((VWF, F5, thrombin reactive protein 1 (THBS1), mannose-binding lectin-associated serine protease 2 (MASP2), apolipoprotein B (APOB), and apolipoprotein (a) (LPA)) were up-regulated and two proteins (Metalloproteinase inhibitor 1(TIMP1), ferritin light chain (FTL)) were down-regulated. The results of this study showed that several proteins involved in complement and coagulation cascades were up-regulated in the serum of patients.

## Introduction

Thymoma is one of the most common tumors in the anterior mediastinum, arising from thymic epithelial cells. The incidence of thymoma is about 2.5 per million people per year^1^. Thymoma is closely related to the neuromuscular disease myasthenia gravis^2^. At present, the pathogenesis of thymoma is not clear^3^. Thymoma detection in the early stages of the disease is essential to improving the prognosis and survival probability. In particular, serum biomarkers are most attractive because simple blood tests are minimally invasive for patients and can provide valuable information about thymoma status. Thymoma-related biomarkers have not yet been identified and used clinically.

The molecular characteristics of different histological and clinical types of thymoma are often used to improve the diagnosis and treatment of thymoma and determine patients' prognoses^4^. Most of the previous studies have performed biomarker searches and tumor analysis for thymoma by comparing differential genes. These studies focused on differences in gene expression between thymoma subtypes and thymic carcinomas^5–9^. Although these studies have identified biomarkers of diagnostic or prognostic significance for thymic epithelial tumors, further validation is needed in clinical practice. The proteomic analysis provides global comparisons of proteins in almost all biological samples, enabling the identification of multiple proteins of interest in a single experiment^10^. Proteins are actual mediators of intracellular processes, and changes in the proteome may more accurately reflect cellular changes. Some studies have classified thymomas by comparing the proteomic characteristics of thymus and thymoma or different types of thymoma^11–13^. However, there is still a lack of research on the serum proteomic characteristics of thymoma, which hinders our understanding and treatment of the tumor.

The analysis of serum protein components in patients with thymoma may provide a basis for an in-depth understanding of the pathogenesis of thymoma and will have far-reaching significance for the research and development of the treatment of thymoma. In this study, the serum protein profiles of people with thymomas and healthy people were quantitatively examined to identify the protein components, revealing varying abundances between the two groups.

## Methods

### Patients

A total of twenty patients with thymoma diagnosed in Shijiazhuang people’s hospital affiliated with Hebei Medical University from February 2018 to May 2019 were chosen as the case group, and nine healthy people served as the control group. Inclusion criteria: (I) over the age of eighteen; (II) thymoma was diagnosed by postoperative pathology; (III) thymoma was classified according to the World Health Organization (WHO) histological classification. Exclusion criteria: (I) under 18 years old; (II) people were suffering from severe cardiac disease, other malignant malignancies, or unable to cooperate. All individuals, including patients and healthy controls, were of Han ethnicity. The aim of the study and the precautions that should be followed was explained to all investigators. Each participant in the study signed a documented informed consent form. The hospital's ethics committee approved the study.

### Preparation of serum samples

Before the operation, the patient drew blood. Patients and healthy participants' fasting venous blood was obtained in the morning. The serum was separated from the blood samples by centrifuging at 12,000*g* for 10 min at 20 °C. The resulting supernatant was stored at − 80 °C for mass spectrometry analysis. The ProteoExtract Albumin/immunoglobulin G (IgG) Removal kit by Merck Millipore (MA, USA) was used to remove albumin and IgG from the serum samples. The serum samples were treated with a reaction solution (1% sodium dodecyl sulfate (SDS), 4.5 mM (mM) dithiothreitol (DTT), 9 mM iodoacetamide (IAA)) and incubated at 55 °C for 30 min for protein denaturation, disulfide bond reduction, and sulfhydryl alkylation. The Bradford assay was used to quantify the protein concentration in samples. In order to digest the sample with enzyme, 1 mg/ml Trypsin N-tosyl-L-phenyl chloromethyl keton (TPCK) was added to the sample at 1:50 and digested overnight at 37 °C. The next day, phosphoric acid was added to stop the enzyme digestion, and the pH of the solution was adjusted to about 3.0. The peptides after enzymatic hydrolysis were desalted by SOLA™ SPE96 orifice plate. Before desalting, adjust the pH value of the sample solution to close to 7. The sample loaded onto the SOLA™ SPE 96 orifice plate using liquid–liquid extraction (LLE) method. The peptides were washed with 200 μL 5% methanol, then eluted with 150 μL methanol and dried in vacuum for MS analysis.

### Data independent acquisition (DIA) analysis

For mass spectrometry (MS) analysis, DIA technology was used. The experiment was carried out using an Agilent 1100 HPLC System and a Q Exactive HF. The first step was to establish a protein map library by using the traditional data dependent acquisition (DDA) method. The DDA mass spectrometry conditions were a MS1 scan (scanning range, 350–1650 m/z; resolution, 120 K; automatic gain control (AGC), 3 × 10^6^; maximum injection time, 100 ms) and MS2 scanning (scanning range, 200–2000 m/z; resolution, 30 K; AGC, 2 × 10^5^; max injection time, 80 ms). We identified a total of 898 proteins in the protein library using DDA. The database searching was performed using Spectronaut Pulsar software, with a false discovery rate (FDR) of 1% at both the peptide and protein levels. The following parameters were used for the database search: missed cleavage, 2; fixed modification, carbamidomethyl; variable modification, oxidation. The second step was to use DIA technology to collect the mass spectrum data of each sample. In this experiment, all enzymolysis samples were separated by Agilent Zorbax Extend-C18 in a mobile phase with pH = 10. Both mobile phases were adjusted to a pH value of 10 using ammonia. The elution was performed with a gradient of solvent A (0.1% formic acid aqueous solution) and solvent B (0.1% hydroxyurea, 80% dicarboxamide, and 20% water), starting with 95% A and 5% B, and gradually changing the ratio to 5% A and 95% B over the course of 81 min. The detection wavelength was set at 210 nm, and the enzymolysis peptide of each sample was collected separately. The scanning range was set to 350–1250 m/z, with an isolation window of 26 m/z. The Spectronaut Pulsar processed secondary mass spectrometry files obtained from the DIA scan, which could be utilized for database retrieval. The results were used to create the spectrogram library. The key parameters of the DIA data analysis were as follows: precursor qvalue cutoff, 5%; protein qvalue cutoff, 5%; normalization strategy, local normalization; quantity MS-level, MS2. The quantitative data from the DIA analysis were screened with a 1% false discovery rate (FDR). The normalized data were transformed for difference comparison and t-test analysis to log2 data. The fold change ratio and P value were utilized to identify protein components with differing abundances between patients and the control group. Tests for differential protein were conducted using the ratio and P value. The protein was over-represented if the fold change was ≥ 1.2 and P ≤ 0.05; the protein was under-represented if the fold change was ≤ 0.833 and P ≤ 0.05.

### Bioinformatics analysis

In order to perform functional annotation and enrichment analysis, the Gene Ontology (GO) and Kyoto Encyclopedia of Genes and Genomes (KEGG) database was employed. The STRING database was utilized to evaluate the interaction connection of different proteins. Network analysis was carried out on the gathered data using the Python tool "networkx".

### Statistical analyses

The mean ± standard deviation was used to represent continuous variables. The variations between the case and control groups and the proportion of categorical variables were compared. The chi-square test or Fisher test was used to compare the two groups' categorical variables. All statistical analyses were performed using SPSS Version 24 (Chicago, IL, USA) and R 4.1.0 (https://www.r-project.org/). P ≤ 0.05 was regarded as statistically significant.

### Ethical approval

The Ethics Committee of People’s Hospital of Shijiazhuang has given this study its ethical approval (Approval number 201894). All of the patients gave their informed consent and were approved by the ethics committee. All methods were performed in accordance with the Declaration of Helsinki.

## Results

### Patient basic information

Primary data for each patient and healthy participant in the study are shown in Table 1. The thymoma group consisted of twelve males. This group's average age was 47.38 ± 8.18. The average body mass index (BMI) of thymoma patients was 23.27 ± 4.44. The control group included four men. The control group's average age was 40.54 ± 8.98. The average BMI in the control group was 22.78 ± 3.05. There were no significant age, gender, or BMI variations between the two groups (P > 0.05).

**Table 1:** Baseline characteristics of patients included in the study.

Variables	Non-thymoma (n = 9)	Thymoma (n = 20)	*P v*alue
Age (mean (SD), years)	40.54 (8.98)	47.38 (8.18)	0.053
Gender (n, %)			
Female	5 (55.56)	8 (40.00)	0.707
Male	4 (44.44)	12 (60.00)	
BMI (mean (SD))	22.78 (3.05)	23.27 (4.44)	0.774

*SD* standard deviation.

### Proteomics analysis

**Figure 1:**
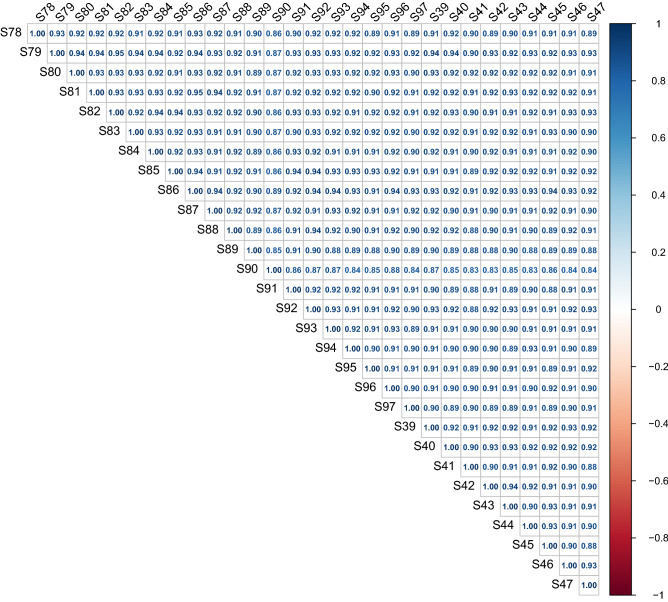
The heat map presents the results of a quantitative correlation analysis between proteins across multiple samples. The color intensity of each cell in the picture represents the intensity and direction of the correlation between protein levels in a given sample. Blue is a positive correlation and red is a negative correlation. The darker the color is, the stronger the correlation is. The software used to create the map is R 4.1.0 (https://www.r-project.org/).

**Figure 2:**
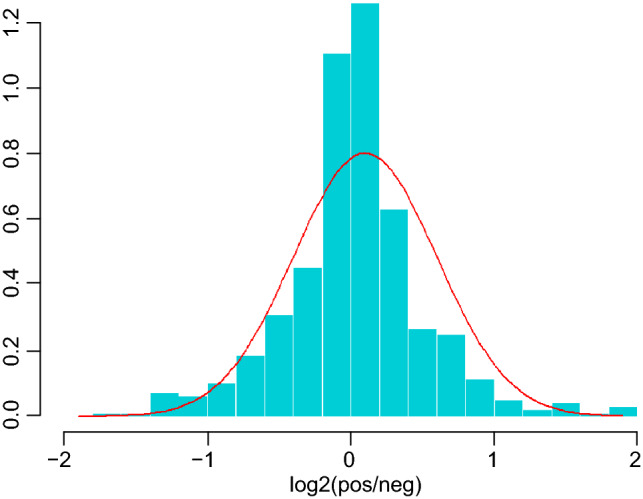
Histone ratio distribution of cases. The ratio distribution of the case group is roughly normal. It means that the protein levels have a high concentration around the mean value and the concentration decreases as the values move further away from the mean.

**Figure 3:**
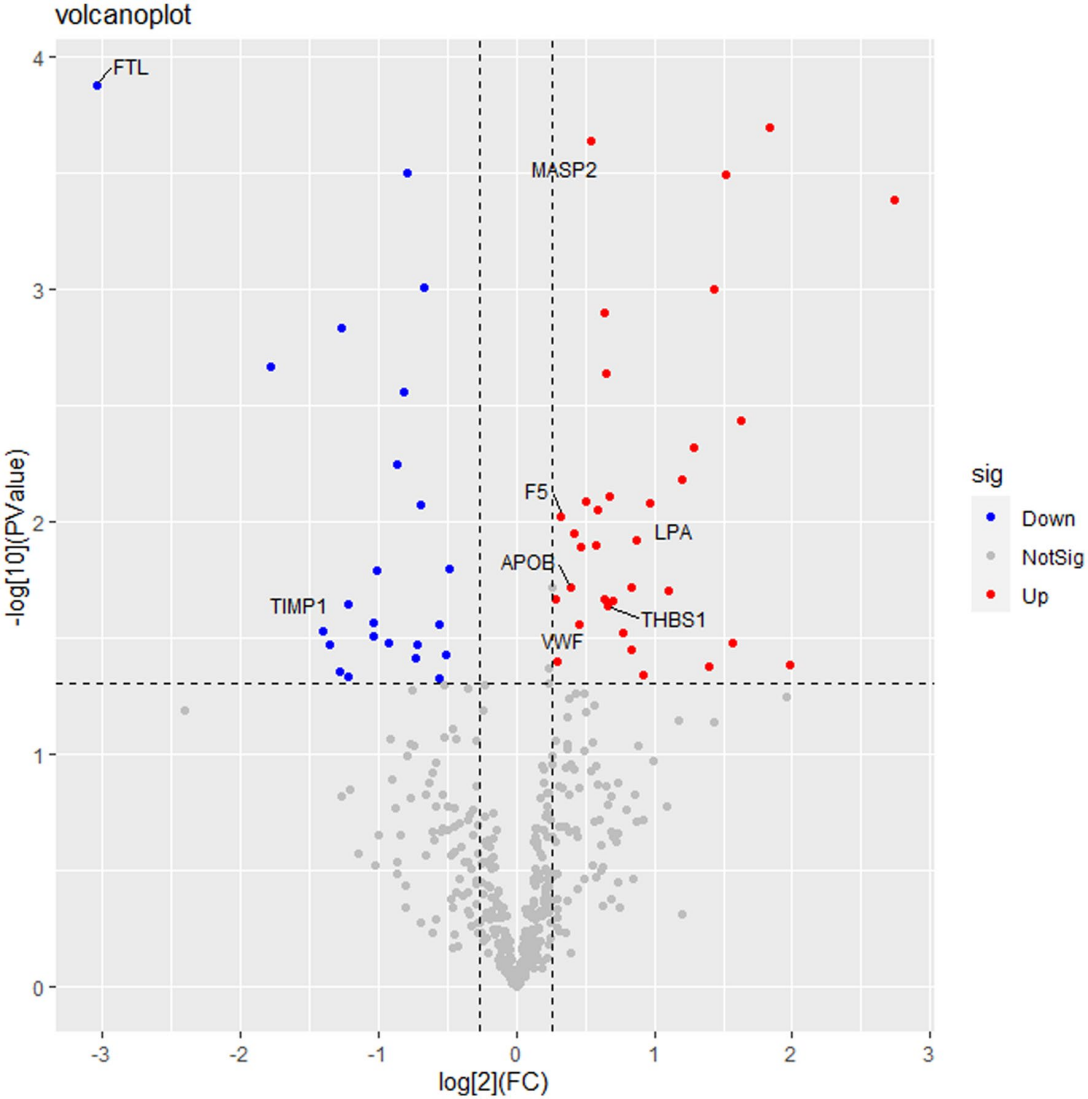
Volcano figure. The main differential proteins are marked in the figure. Blue dots on the plot represent down-regulated proteins, red dots represent up-regulated proteins, and gray dots represent proteins that are not significantly differentially expressed.

**Figure 4:**
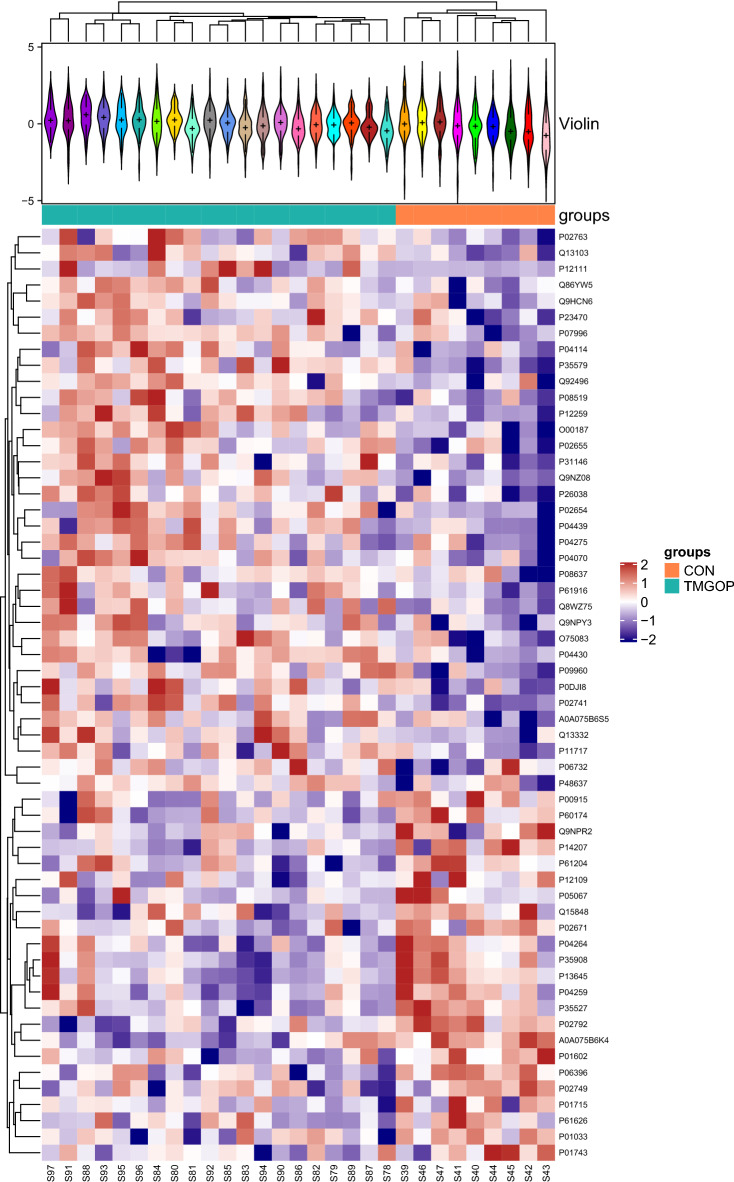
Clustering heat map of all proteins. At the top is a violin diagram, a combination of a box and density diagram, indicating the probability distribution of the expression value of the sample. A flatter violin indicates a more concentrated data. Different color fillings represent different samples. The median of the data is represented by the "+" symbol in the middle, and the vertical axis shows the protein expression level. Below the violin is a column annotated heat map, where samples of the same group are represented by blocks of the same color. The clustering heat map below organizes proteins based on expression levels, with red indicating high expression and blue representing low expression, each row representing protein expression across different groups. The software used to create the map is R 4.1.0 (https://www.r-project.org/).

486 serum proteins were found in all groups, and Table 2 summarizes the quantitative data for all of these proteins. The correlation between the protein levels and the samples was strong as evidenced by the correlation coefficients, which were all above 0.8 (Fig. 1). The ratio distribution in the case group is essentially normal (Fig. 2). Fifty-eight proteins were differentially expressed across the two groups, with 35 being up-regulated and 23 being down-regulated (Table 2, Figs. 3 and 4).

**Table 2:** Differential protein information.

Protein names	Protein description	Log2FC	FC (pos/neg)	*P *value	State
IGLV3-10_HUMAN	Immunoglobulin lambda variable 3–10	− 0.79736	0.5754	0.000316	Down
CA1_HUMAN	Carbonic anhydrase 1	− 1.04011	0.486292	0.031199	Down
TIMP1_HUMAN	Metalloproteinase inhibitor 1	− 1.4016	0.37851	0.029625	Down
IGKV1-5_HUMAN	Immunoglobulin kappa variable 1–5	− 0.69429	0.618013	0.008455	Down
IGLV3-1_HUMAN	Immunoglobulin lambda variable 3–1	− 1.35679	0.390449	0.033893	Down
IGHV1-46_HUMAN	Immunoglobulin heavy variable 1–46	− 0.50698	0.703696	0.037431	Down
FGA_HUMAN	Fibrinogen alpha chain	− 0.48546	0.714271	0.015975	Down
APOH_HUMAN	Beta-2-glycoprotein 1	− 0.55666	0.679876	0.047006	Down
FTL_HUMAN	Ferritin light chain	− 3.04528	0.121138	0.000134	Down
KRT6B_HUMAN	Keratin, type II cytoskeletal 6B	− 0.92139	0.528	0.033326	Down
KRT1_HUMAN	Keratin, type II cytoskeletal 1	− 0.72761	0.603903	0.03893	Down
APP_HUMAN	Amyloid-beta precursor protein	− 1.21616	0.430426	0.022811	Down
GSN_HUMAN	Gelsolin	− 0.67425	0.626656	0.000986	Down
COL6A1_HUMAN	Collagen alpha-1(VI) chain	− 1.2767	0.412739	0.044503	Down
KRT10_HUMAN	Keratin, type I cytoskeletal 10	− 1.27342	0.413678	0.001474	Down
FOLR2_HUMAN	Folate receptor beta	− 1.78131	0.29092	0.002167	Down
KRT9_HUMAN	Keratin, type I cytoskeletal 9	− 0.8124	0.569435	0.002781	Down
KRT2_HUMAN	Keratin, type II cytoskeletal 2 epidermal	− 0.86363	0.549568	0.005695	Down
TPI1_HUMAN	Triosephosphate isomerase	− 1.00739	0.497446	0.016195	Down
ARF3_HUMAN	ADP-ribosylation factor 3	− 1.03295	0.488708	0.027111	Down
LYZ_HUMAN	Lysozyme C	− 0.71359	0.609802	0.033574	Down
ADIPOQ_HUMAN	Adiponectin	− 0.55423	0.681019	0.027705	Down
SEMA4B_HUMAN	Semaphorin-4B	− 1.21776	0.429949	0.046452	Down
IGKV1-27_HUMAN	Immunoglobulin kappa variable 1–27	0.592905	1.508281	0.008978	Up
MASP2_HUMAN	Mannan-binding lectin serine protease 2	0.536809	1.45076	0.000232	Up
WDR1_HUMAN	WD repeat-containing protein 1	1.289225	2.443968	0.004831	Up
APOC1_HUMAN	Apolipoprotein C-I	0.421383	1.339211	0.011336	Up
APOC2_HUMAN	Apolipoprotein C-II	0.640782	1.559174	0.001268	Up
CRP_HUMAN	C-reactive protein	1.522919	2.873719	0.000325	Up
ORM1_HUMAN	Alpha-1-acid glycoprotein 1	0.413798	1.332188	0.011337	Up
PROC_HUMAN	Vitamin K-dependent protein C	0.282106	1.215969	0.021412	Up
APOB_HUMAN	Apolipoprotein B-100	0.391444	1.311705	0.019289	Up
VWF_HUMAN	von Willebrand factor	0.450121	1.366155	0.02761	Up
IGKV1-16_HUMAN	Immunoglobulin kappa variable 1–16	1.397068	2.633659	0.041719	Up
HLA-A_HUMAN	HLA class I histocompatibility antigen, A alpha chain	0.967895	1.955985	0.008303	Up
CKM_HUMAN	Creatine kinase M-type	1.564804	2.958372	0.033213	Up
THBS1_HUMAN	Thrombospondin-1	0.659314	1.579332	0.023144	Up
LPA_HUMAN	Apolipoprotein(a)	0.87376	1.832433	0.012022	Up
FCGR3A_HUMAN	Low affinity immunoglobulin gamma Fc region receptor III-A	0.917776	1.889201	0.045687	Up
LTA4H_HUMAN	Leukotriene A-4 hydrolase	1.434206	2.702334	0.000999	Up
SAA1_HUMAN	Serum amyloid A-1 protein	0.6345	1.5524	0.021592	Up
IGF2R_HUMAN	Cation-independent mannose-6-phosphate receptor	0.832351	1.780585	0.035688	Up
COL6A3_HUMAN	Collagen alpha-3(VI) chain	1.980825	3.947188	0.041069	Up
F5_HUMAN	Coagulation factor V	0.325533	1.253128	0.009494	Up
PTPRG_HUMAN	Receptor-type tyrosine-protein phosphatase gamma	0.30186	1.232732	0.039862	Up
MSN_HUMAN	Moesin	0.650774	1.570011	0.002293	Up
CORO1A_HUMAN	Coronin-1A	1.631035	3.09735	0.003689	Up
MYH9_HUMAN	Myosin-9	1.835409	3.568725	0.000203	Up
GSS_HUMAN	Glutathione synthetase	0.775263	1.711502	0.029976	Up
NPC2_HUMAN	NPC intracellular cholesterol transporter 2	0.472182	1.387206	0.012829	Up
SPP2_HUMAN	Secreted phosphoprotein 24	0.582264	1.497196	0.012684	Up
PTPRS_HUMAN	Receptor-type tyrosine-protein phosphatase S	1.198473	2.294966	0.006589	Up
TREML1_HUMAN	Trem-like transcript 1 protein	0.678363	1.600323	0.007836	Up
ROBO4_HUMAN	Roundabout homolog 4	0.498242	1.412491	0.008143	Up
CFHR4_HUMAN	Complement factor H-related protein 4	0.836601	1.785838	0.019324	Up
GP6_HUMAN	Platelet glycoprotein VI	1.107955	2.155398	0.019723	Up
CD93_HUMAN	Complement component C1q receptor	0.697005	1.621136	0.021892	Up
ERAP1_HUMAN	Endoplasmic reticulum aminopeptidase 1	2.740361	6.682373	0.000416	Up

### GO functional annotation and enrichment analysis

**Figure 5:**
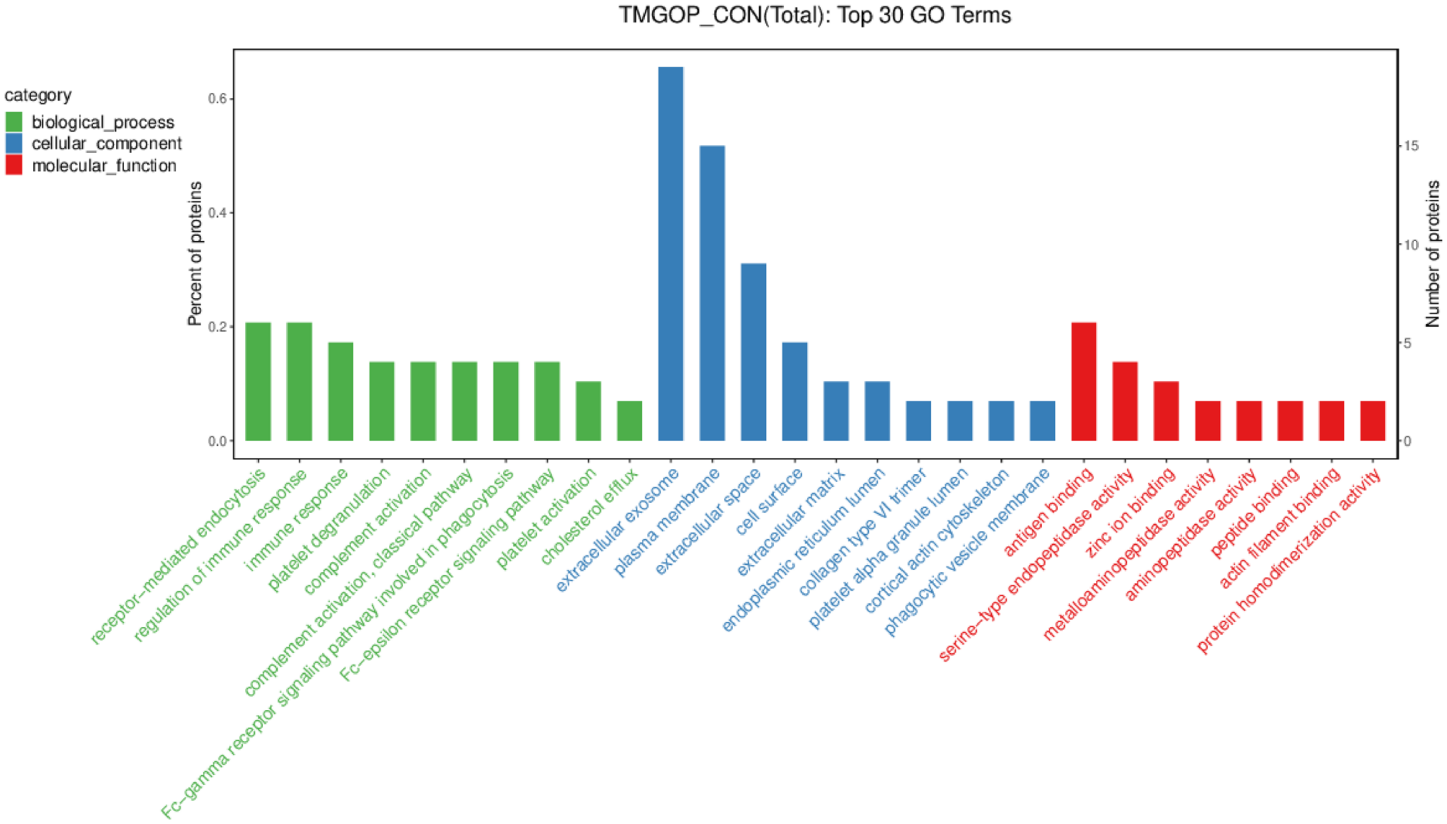
GO annotation and enrichment analysis. In the figure, the x-coordinate represents the name of the GO entry, while the y-coordinate shows the number of proteins and their percentage in relation to the corresponding entry.

Three GO levels, including the biological process (BP), cellular component (CC), and molecular function (MF), were used to categorize differently regulated proteins (Fig. 5). Protein differences in the patient group for the BP category were primarily involved in receptor-mediated endocytosis (7.79%), immunological response (6.49%), and immune response control (7.79%). The extracellular exosome accounted for 20.88% of the CC, the serum membrane for 16.48%, and the extracellular space for 9.89%. In the MF category, 26.09% played a role in antigen binding, 17.39% were serine-type endopeptidase activity, and 13.04% had a zinc ion binding function.

### KEEG functional annotation and enrichment analysis

**Figure 6:**
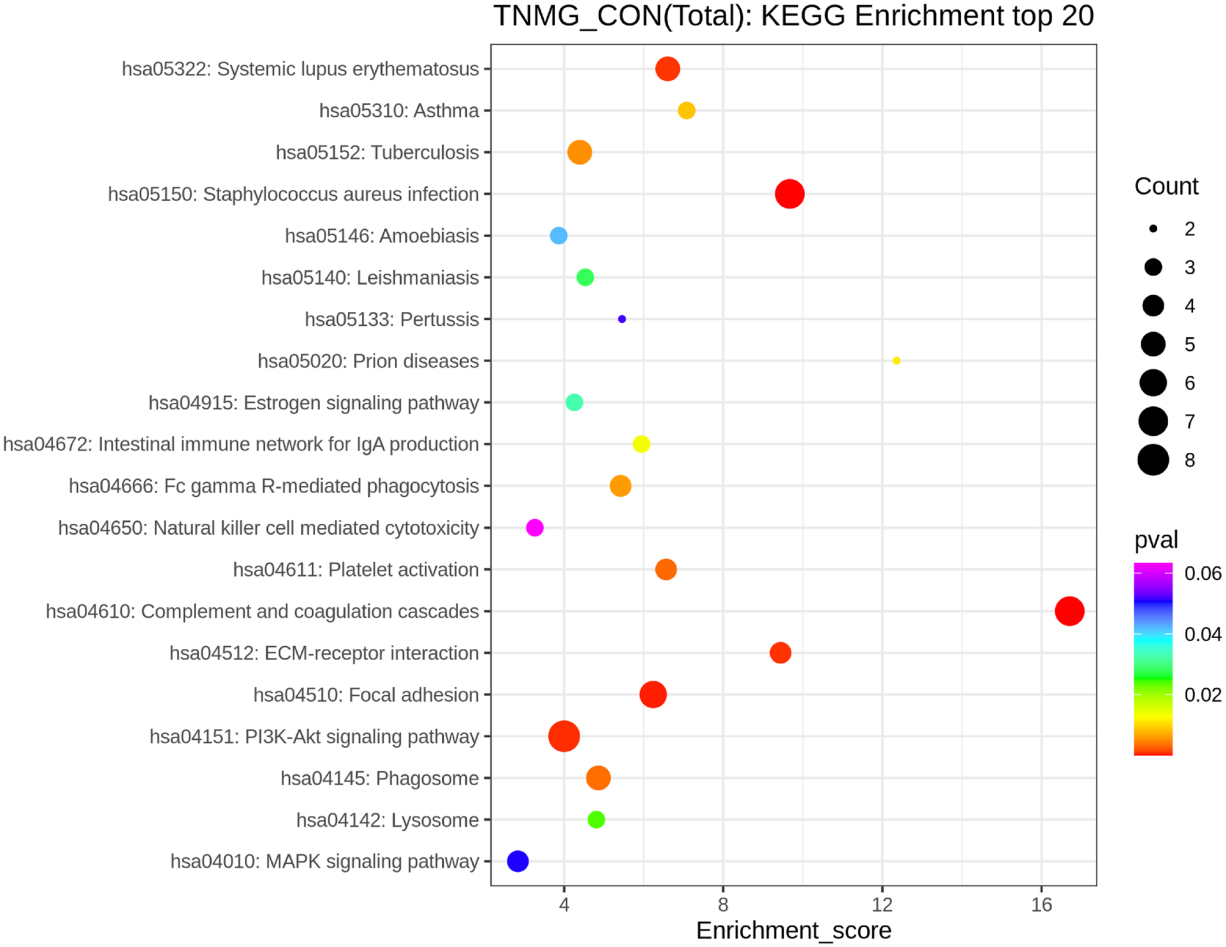
KEGG annotation and enrichment analysis. In the figure, the x-axis Enrichment score is the enrichment score and the y-axis is the pathway information of top20. The larger the bubble, the more the number of differential proteins, the color of the bubble changed from red–green–blue-purple, and the smaller the enrichment p value was, the greater the significance was.

**Figure 7:**
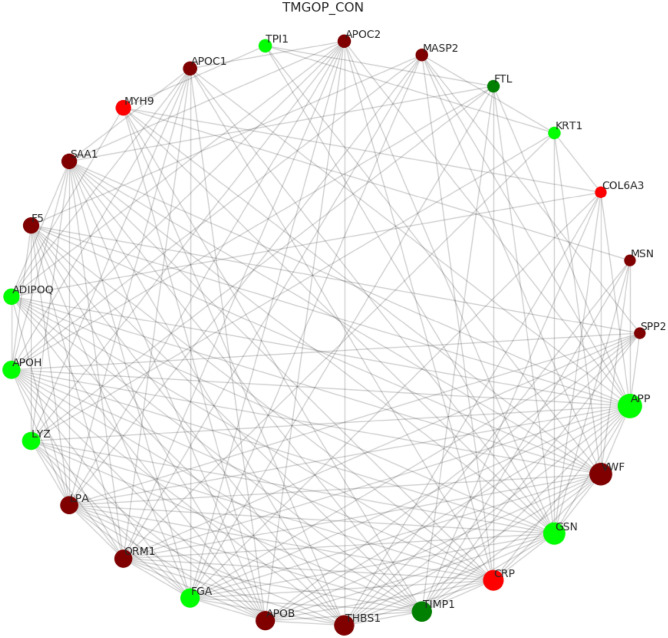
Enrichment KEGG pathway diagram. In the picture, the circle represents the differential protein, the red represents the up-regulated protein, and the green represents the down-regulated protein; the size of the circle represents the degree of connectivity, and the larger the circle, the higher the connectivity.

We performed a KEGG pathway analysis on the identified differential proteins to better understand their involvement in healthy or pathological activities and determine the metabolic and signaling pathways involved^14^. According to our findings, the differential proteins primarily engage in the following biological processes: complement and coagulation cascades, phosphoinositide 3-kinase (PI3K)/protein kinase B (AKT) signaling pathway, and staphylococcus aureus infection. (Fig. 6). Complement and coagulation cascades have the highest KEEG enrichment^15^ (Fig. 7).

### PPI network construction

**Figure 8:**
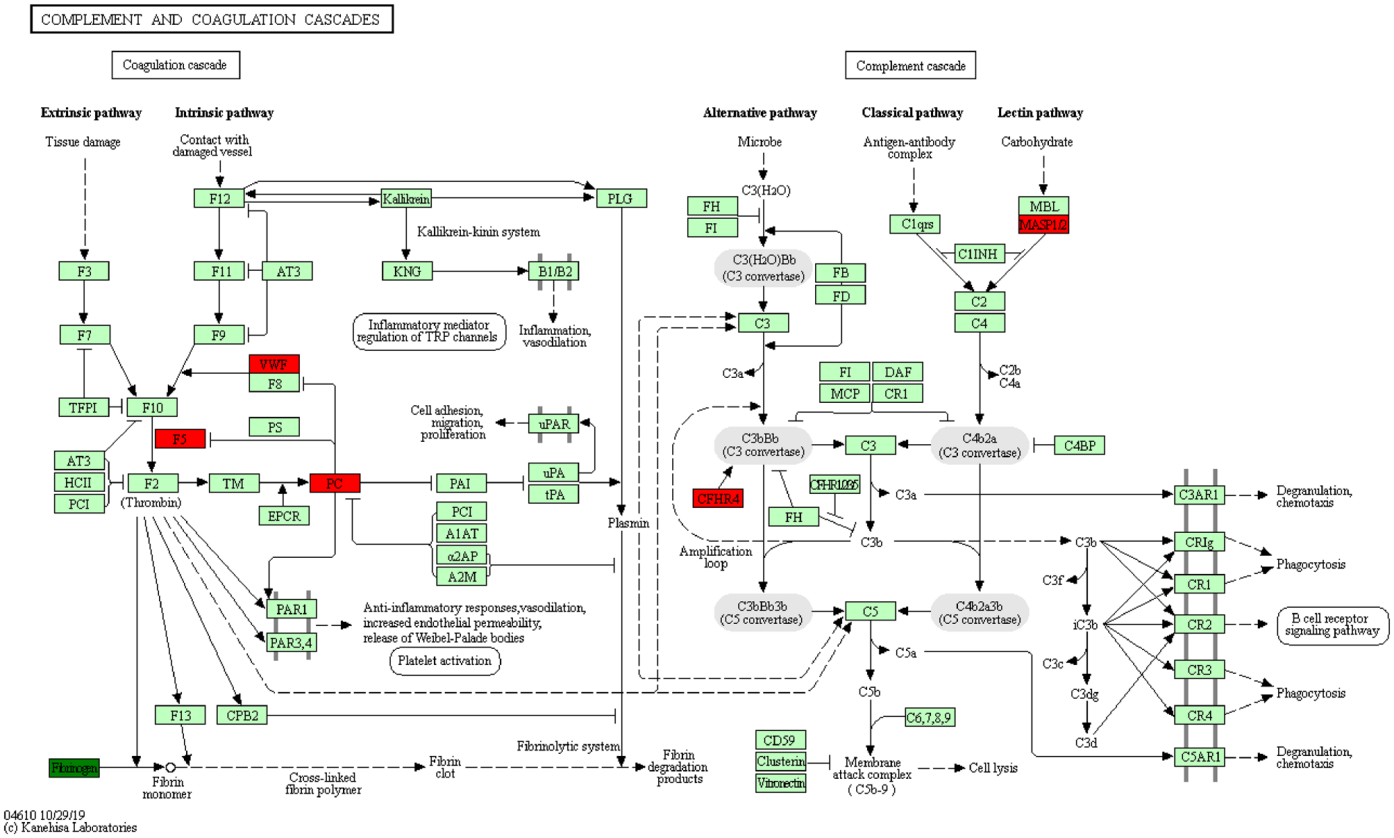
Protein interaction network analysis. The rectangle represents the enzyme/gene, the red indicates the up-regulated protein, the green indicates the down-regulated protein, and the yellow indicates both up-regulated and down-regulated protein. The colorless rectangle represents the genes present in the map, the light green rectangle represents the species-specific genes, and the light purple rectangle represents the genes that exist in both map and ko. KEGG Pathway (KEGG, 2021).

The string database was used to assess the interaction of different proteins, and the nodes with the top 25 node connectivity were visualized by the python package ‘networkx’, which was displayed by protein identification (ID) and gene name, respectively (Fig. 8). In the picture, the circle represents the differential protein/gene, the red represents the up-regulated protein/gene, and the green represents the down-regulated protein/gene; the circle's size shows the degree of connectivity; the larger the circle, the greater the connectivity.

Overexpressed proteins with significant changes are depicted in the figure, including von willebrand factor (VWF), thrombin reactive protein 1 (THBS1), coagulation factor V (F5), and mannose-binding lectin-associated serine protease 2 (MASP2), apolipoprotein B (APOB), and apolipoprotein (a) (LPA). The expression of two proteins, Metalloproteinase inhibitor 1 (TIMP1) and ferritin light chain (FTL) was significantly insufficient.

## Discussion

Thymoma epithelial cells have endocrine activity^16^. Some reports on diagnostic or prognostic proteins in thymoma have been published in recent years. Sasaki et al. found that serum periosteal levels may indicate tumor invasion and progression of thymoma^17^. The results of Du et al. show that Mucin 1 (MUC1) and glucose transporter type 1 (GLUT1) staining are helpful in the differential diagnosis of thymic carcinoma and B3 thymoma with high sensitivity and specificity^18^. However, there are few systematic studies on the changes in serum protein components in patients with thymoma. This study is the first to conduct a global proteome map analysis using serum and liquid chromatography-mass spectrometry (LC–MS)/MS from thymoma and healthy controls. In this study, 58 distinct proteins were found in the serum samples of thymoma patients, of which 35 were overexpressed in the patient samples, and 23 were underexpressed compared with the control samples.

According to GO functional annotation, most of these proteins are factors involved in immunoreactive regulatory antigen binding in exosomes and serum membranes. These distinct proteins were predominantly connected to coagulation, complement system activation, and the PI3K-Akt signal pathway, according to KEGG functional annotation. It is worth noting that the members of the complement and coagulation cascade pathway have been enriched, which proved that VWF, F5, THBS1 and MASP-2 were overexpressed in patient samples.

The prothrombotic condition seen in cancer patients is frequently caused by an imbalance between the coagulation and fibrinolysis systems^19–21^. Many procoagulant factors, such as tissue factors and cancer procoagulants, are secreted or expressed on the cell surface of many tumors. VWF is considered to mediate tumor cell attachment to platelets and facilitate their systemic dissemination. The vascular endothelial growth factor (VEGF) increases the proliferation and differentiation of vascular endothelial cells (the principal producer of VWF) during angiogenesis, which happens during tumor growth, resulting in higher serum VWF levels in cancer patients^22^. This study showed that serum VWF levels in thymoma patients were higher than in healthy controls.

In addition, F5 also increased in the same pathway. F5 is a circulating high molecular weight (330 KDa) factor, which plays an essential role in the coagulation cascade reaction. When F5 is activated, it acts as a cofactor to activate factor X, which converts prothrombin into thrombin^23^. F5 gene polymorphism is associated with the risk of breast cancer, colorectal cancer, gastric cancer and other cancers^24,25^. THBS is a multifunctional extracellular matrix protein family involved in tissue remodelling and has been linked to cancer, wound healing, and embryonic development^26^. The first member to be identified is THBS1, which inhibits endogenous angiogenesis. THBS1 binds to several cellular receptors to fulfil various tasks^27^. According to earlier research, THBS1 is abundantly expressed in a variety of tumor types and encourages tumor cell adhesion, proliferation, apoptosis, invasion, and metastasis^28–31^.

The complement-activated mannan-binding lectin (MBL) pathway is an inherent immune protection mechanism that reacts to infections directly. MBL and MBL-related serine proteases circulate in the blood. MASP-2 is a protease that activates complement cascades. The binding of MBL initiates the complement-activated MBL pathway to carbohydrates present on the surface of microorganisms. This leads to the activation of C4 and C2 mediated by MASP-2 to produce C3 invertase (C4bC2b), which leads to the neutralization of pathogens^32–35^. High MASP-2 concentration is associated with poor prognosis in patients with colorectal cancer^36^. In addition, the MASP-2 gene is also highly associated with lung and endometrial cancer^37,38^. MASP-2 expression was higher in patients with lung adenocarcinoma than in people with lung squamous cell carcinoma^39^.

In this study, the serum TIMP-1 of patients with thymoma was lower than that of normal controls. TIMPs were first discovered as a serum protein linked to the formation of erythroid progenitor cells and the inhibition of collagenase^40^. TIMPs are tissue-specific proteins that block matrix metalloproteinases (MMPs). MMPs are proteolytic enzymes that break down basement membrane and extracellular matrix^41^. The increased expression of MMPs in various cancers is associated with tumor aggressiveness. Metallopeptidase inhibitors (TIMPs) are negative regulators of tumor invasion and metastasis. However, the role of TIMP-1 in cancer is controversial because it can have both pro- and antitumoral effects. In addition to inhibiting matrix metalloproteinases, TIMP-1 can also promote cell growth and proliferation and inhibit cell apoptosis, which may be involved in the regulation of angiogenesis. Clinical investigations have demonstrated a strong correlation between high levels of TIMP-1 expression and poor prognosis or tumor growth in various malignancies, including lung cancer, brain prostate cancer, breast cancer, colon cancer, and others^42–48^. Yukiue et al. found that the expression level of TIMP-1 was increased in invasive thymoma tissues and speculated that TIMP-1 and MMP-1 played an essential role in the invasion process of thymoma^49^. In studies of cellular events associated with TIMP-induced cell growth, TIMP-1 was found to stimulate the Rat sarcoma viral oncogene homolog (RAS) protein activation^50^. RAS protein is the upstream regulator of the Tyrosine Kinase/Mitogen-Activated Protein Kinase (TyK/MAPK) cascade. In other words, TIMP-1 can activate the tyrosine kinase/mitogen-activated protein kinase signal pathway. The location of TIMP-1 may hold the key to understanding its dual function: intracellular TIMP-1 boosts cancer cell proliferation and survival by interfering with various signal pathways, whereas extracellular TIMP-1 is restricted to the extracellular matrix to prevent metastasis by inhibiting nearby MMPs. It can be speculated that TIMP-1 inhibits invasion and metastasis mainly by inhibiting the remodelling of the extracellular matrix in thymoma. Further research is needed to confirm this.

This research has certain limitations. First, the study's sample size was limited, which may have resulted in selection bias. Secondly, the differential proteins identified were not confirmed by other detection methods. As a result, if conditions permit, subsequent investigations with bigger sample sizes may be done, and other analytical techniques will be required to confirm the different proteins found.

## Conclusion

In the patient's serum, several complement and coagulation-related proteins were up-regulated. These serum proteins may become serological biomarkers of thymoma in the future. Extracellular TIMP-1 of thymoma may prevent invasion and metastasis mainly by inhibiting the remodelling of the extracellular matrix.

## Data Availability

The data will be accessible by contacting the corresponding author of this study.
